# Effect of robot-assisted gait training on improving cardiopulmonary function in stroke patients: a meta-analysis

**DOI:** 10.1186/s12984-024-01388-9

**Published:** 2024-05-30

**Authors:** Xiao Chen, Lu Yin, Yangbo Hou, Jie Wang, Yongyi Li, Juntao Yan, Jiming Tao, Shujie Ma

**Affiliations:** 1 Department of Rehabilitation Medicine, Shuguang Hospital Affiliated to Shanghai University of Traditional Chinese Medicine, Shanghai, China; 2 Department of Rehabilitation Medicine, Yueyang Hospital of Integrated Traditional Chinese and Western Medicine, Shanghai University of Traditional Chinese Medicine, Shanghai, China; 3 Department of Neurology, Putuo Hospital, Shanghai University of Traditional Chinese Medicine, Shanghai, China; 4 Rehabilitation department of traditional Chinese Medicine, The Second Rehabilitation Hospital of Shanghai, Shanghai, China; 5 Department of Tuina, Yueyang Hospital of Integrated Traditional Chinese and Western Medicine, Shanghai University of Traditional Chinese Medicine, Shanghai, China

**Keywords:** Robot-assisted gait training, Cardiopulmonary fitness, Stroke, 6MWT, Meta-analysis

## Abstract

**Objective:**

Understanding the characteristics related to cardiorespiratory fitness after stroke can provide reference values for patients in clinical rehabilitation exercise. This meta- analysis aimed to investigate the effect of robot-assisted gait training in improving cardiorespiratory fitness in post-stroke patients, compared to conventional rehabilitation training.

**Methods:**

PubMed, EMBASE, Web of Science, Cochrane Database of Systematic Reviews, CBM, CNKI and Wanfang databases were searched until March 18th, 2024. Randomized controlled trials (RCTs) comparing the effectiveness of robot-assisted gait training versus control group were included. The main outcome variable was peak oxygen uptake. 6-minute walking test, peak heart rate, peak inspiratory expiratory ratio as our secondary indicators. RevMan 5.3 software was used for statistical analysis.

**Results:**

A total of 17 articles were included, involving 689 subjects. The results showed a significant effect for robot-assisted gait training to improve VO_2peak_ (MD = 1.85; 95% CI: -0.13 to 3.57; *p* = 0.04) and 6WMT (MD = 19.26; 95% CI: 10.43 to 28.08; *p* < 0.0001). However, no significant difference favouring robot-assisted gait training were found in HR_peak_ (MD = 3.56; 95% CI: -1.90 to 9.02; *p* = 0.20) and RER_peak_ (MD = -0.01; 95% CI: -0.04 to 0.01; *p* = 0.34).

**Conclusion:**

These results showed that robot-assisted gait training may have a beneficial effect in improving VO_2peak_ and 6WMT, with a moderate recommendation level according to the GRADE guidelines.

**Supplementary Information:**

The online version contains supplementary material available at 10.1186/s12984-024-01388-9.

## Introduction

The motor dysfunction of stroke patients reduces their aerobic exercise ability [1], while the significant decline in cardiopulmonary function adaptability after stroke leads to secondary cardiovascular problems in most patients, which further affects their cardiopulmonary function and glucose and lipid metabolism ability, resulting in a vicious cycle of disability [2, 3]. Studies have found that the peak oxygen uptake (VO2peak) of stroke patients is only equivalent to 60% of the standard sedentary value of healthy adults of the same age and sex [4], and the exercise endurance and aerobic capacity are significantly reduced, which is not enough to meet the needs of independent living [5]. However, for about 75% of stroke patients, this early and sustained decline in aerobic capacity delays or inhibits participation in therapeutic exercise programs, leads to difficulties in the post-stroke rehabilitation process and long-term care, and limits an individual’s ability to perform functional activities independently, so that stroke survivors are more likely to be disabled by related heart disease than by the stroke itself [6, 7].

Rehabilitation is an important part of stroke treatment. Traditional rehabilitation strategies for stroke survivors focus on primary nerve injury and aim to restore motor function and reduce injury, including restoring muscle strength and improving motor coordination and control. Conventional rehabilitation therapy usually does not pay enough attention to the improvement of aerobic capacity, and the intensity and training time of physical or occupational therapy activities are not enough to produce cardiopulmonary training effects [8]. Studies have shown that aerobic exercise can improve the VO_2peak_ and walking endurance of stroke patients with mild to moderate injury at the early stage, and their balance ability, exercise ability, cognition and emotion are positively correlated with cardiopulmonary fitness (CRF), which affects the survival rate after stroke [9, 10].

A meta-analysis by Stoller et al. [11] concluded that cardiovascular exercise during the subacute phase of stroke can significantly improve aerobic capacity, walking speed, and walking endurance. However, for patients with early stroke who cannot walk, robot-assisted gait training can partially or completely support the body load, and high-intensity and complex gait cycle training can be conducted for patients who cannot walk, reducing the workload of therapists and improving the accuracy of step length [12]. Recent studies [13–15] have shown that robot-assisted gait training is superior to traditional rehabilitation therapy in improving cardiorespiratory function in stroke patients. Therefore, this study aims to comprehensively evaluate the effectiveness and safety of robot-assisted gait training in improving the cardiopulmonary function of stroke patients through a comprehensive quantitative evaluation and analysis of the literature, and improve the efficacy of robot-assisted gait training treatment and the quality of clinical research.

## Methods

### Protocol and registration

The meta-analysis was conducted according to the recommendations of the Preferred Reporting Items for Systematic Reviews and Meta-Analysis (PRISMA) guidelines [16]. Article searches and data extraction were independently performed by two investigators (CX and HYB), and any discrepancies were resolved by discussion or consensus with a third author (MSJ). The protocol was registered at the International Prospective Register of Systematic Reviews (PROSPERO: CRD42022298786).

### Information sources and search

All relevant studies in the PubMed, EMBASE, Web of Science, Cochrane Database of Systematic Reviews, CBM, CNKI and Wanfang databases were systematically searched. Publication dates ranged from the database inception to March 18th, 2024 to identify relevant studies. The PubMed, EMBASE, Web of Science, Cochrane Database of Systematic Reviews draw up search strategies based on the principles of population, intervention, control, outcome and study design (PICOS). For the other electronic databases, the search approach was adjusted as required. The detailed search strategy for the database used will be provided in the appendix 1. No filters were applied, and language was not restricted. Study selection was based on an initial screening of identified abstracts or titles and a second screening of full-text articles. The reference lists of relevant review articles and meta-analyses were examined to identify other potentially eligible studies.

### Eligibility criteria

Study screening was conducted according the PICOS principle (P-population; I-intervention; C-comparison; O-outcome and S-study design). All trials that met the following criteria were included in this meta-analysis: ① Research design: Randomized controlled trial to explore the effect of gait training on cardiopulmonary function after stroke. ② Subjects: Stroke patients aged > 18 years, meeting the diagnostic criteria of any cerebrovascular disease. ③ Intervention measures: The control group was given routine rehabilitation treatment or routine gait training, and the experimental group was given robot-assisted gait training of the lower limbs, not treadmill gait training. ④ Outcome indicators: The extracted outcome indicators had to include complete outcome data related to cardiopulmonary function after stroke, such as VO_2peak_, 6MWT, HR_peak_, etc.

### Data collection and extraction

The studies that met the search strategy were imported into EndNote X9 for data management, and duplicate articles were removed. Two investigators (CX and HYB) independently preliminarily screened the articles by reading the title and abstract according to the inclusion criteria. Then, the full text was read to eliminate unqualified studies, and finally, the relevant data of the studies were extracted, including basic information (e.g., authorship, publication date, study design, country), participant characteristics (e.g., mean age, sex, sample size, course of disease, mean disease duration), intervention measures (e.g., intervention time, intervention frequency, intervention content, intervention duration), and outcome indicators (e.g., VO_2peak_, 6MWT, HR_peak_, RER_peak_). The primary authors were contacted when relevant data were not reported. In case of disagreement, two investigators discussed and asked the third researcher (MSJ) for advice and finally reached an agreement.

In this study, we used VO_2peak_ as our primary outcome measure and 6 WMT, HR_peak_, and RER_peak_ as secondary outcome measures. 6 WMT was to walk as much as possible within six minutes, during which you can rest. After the time is up, the walk distance was measured. VO_2peak_ refereed to the maximum oxygen uptake of the body at maximum exercise intensity. RER_peak_ refereed to the ratio of carbon dioxide emissions to oxygen consumption per minute. During the exercise test, VO_2peak_, HR_peak_, and RER_peak_ could be measured at maximal effort.

### Risk of bias

Two authors (CX and MSJ) used the Cochrane Risk of Bias tool (RoB 2.0) to evaluate the methodological quality of the included literature. Disagreements were discussed or resolved by the third author (YL). Evaluation the risk of bias across five domains [17]: ① randomization process; ② deviations from intended interventions; ③missing outcome data; ④ measurement of the outcome; ⑤ selection of reported results. The low risk of bias, some concerns and high risk of bias of each item in the study were judged, and the quality of the studies was classified. If all five domains were rated as low risk, the “overall risk” was rated as “low risk of bias”. If at least one domain was rated as some concerns, it’s considered “some concerns”. If at least one domain was rated as high risk of bias, it’s considered “high risk of bias”.

### Certainty of evidence

To assess the quality of evidence, the Grades of Recommendation Assessment, Development, and Evaluation (GRADE) system was used [18].

### Data synthesis and analysis

All analyses were performed using RevMan software (version 5.3; Cochrane Collaboration, Oxford, UK). For continuous data, the mean difference (MD) and confidence interval (CI) were calculated between pre- and post-treatment, and the effect size was evaluated using a Z test. Meta-analysis of at least two studies was conducted for each outcome indicator to investigate the efficacy of gait training on cardiopulmonary function after stroke. A separate analysis was performed by using the changes evaluated by the scores of the items, which included the VO_2peak_, 6MWT, HR_peak_, and RER_peak_.

Heterogeneity of the integrated results was assessed by the Chi-squared test and I^2^ index, and *p* < 0.05 was considered an indication of a significant difference. Following Niering M et al. [19], heterogeneity can be interpreted as trivial (0 ≤ 40%), moderate (30 ≤ 60%), substantial (50 ≤ 90%), or considerable (75 ≤ 100%). If heterogeneity below 50%, a fixed effects model was used. otherwise, a random effects model was used [20]. Sensitivity analysis was used to assess heterogeneity and was carried out by the removal of one study at a time. Subgroup analysis and publication bias assessment were conducted when necessary.

## Results

### Study selection and characteristics

**Fig. 1:**
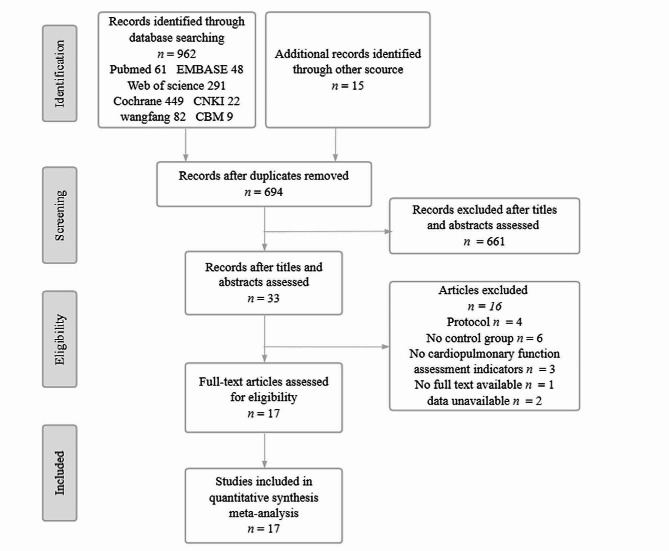
Flow diagram of the eligible study selection process

A total of 962 studies were obtained by searching 7 electronic databases, and 15 supplementary studies were manually tracked, totaling 977 studies. A total of 283 studies remained after duplicates were removed. After preliminary screening by reading the title and abstract, 33 studies remained. After further reading the full text, we had a total of 17 articles that were finally included in the meta-analysis. The study screening process is shown in Fig. 1.

A total of 17 studies were published from 2012 to 2023. A total of 689 study samples were included: 350 in the experimental group and 339 in the control group. The gait training groups all adopted robot-assisted gait training. One study only included male participants [21]. In the control group, one study used treadmill training [22], while routine gait training was reported in another eight studies [23–30], Other studies used routine rehabilitation therapy [13, 14, 21, 31–35]. Most of the subjects were in the subacute phase (two weeks to six months). Four studies involved investigations after six months of stroke [21, 22, 27, 28] and another study involved investigating patients with acute stroke (within 48 h after stroke) [14]. The intervention time ranged from 20 min to 60 min, most of which lasted for 30 min. The intervention frequency of two studies was twice a day [25, 32] and that of other studies was once a day [13, 14, 21–24, 26–30, 33–35]. The intervention period ranged from 2 to 6.5 weeks, and two of the studies were followed up for 8–12 weeks [14, 22]. The details of the main outcome indicators are reflected in the characteristics in Tables 1 and 2 of the appendix 2.

### Study outcomes of peak oxygen uptake

**Fig. 2:**
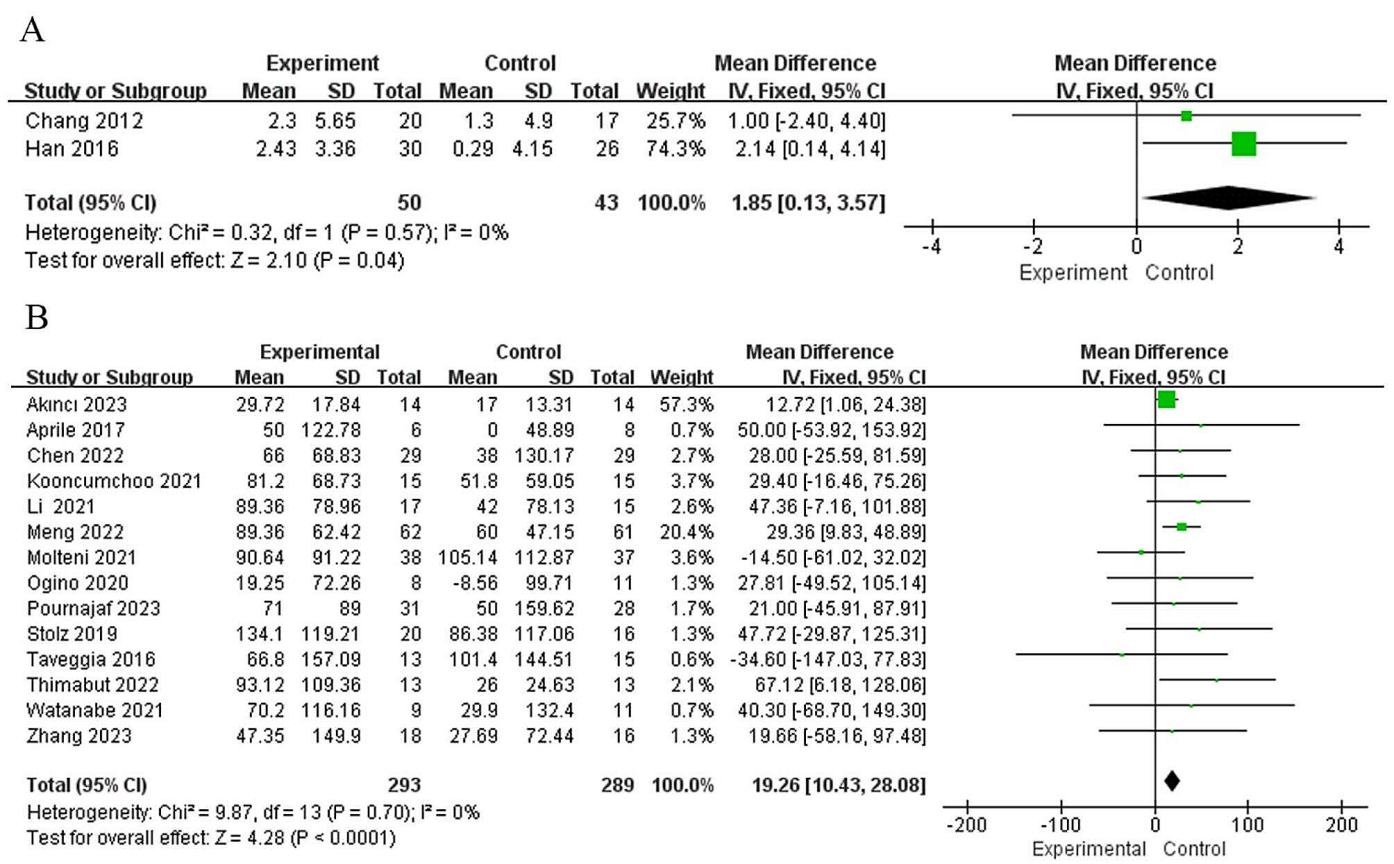
(**A**) Forest plots of meta-analysis to evaluate the effects of robot-assisted gait training on VO_2peak_; (**B**) Forest plots of meta-analysis to evaluate the effects of robot-assisted gait training on 6 WMT

This meta-analysis included two studies [31, 35] that compared the VO_2_peak of 93 patients after intervention. The combined results showed that there was no heterogeneity between the studies (I^2^ = 0%, *p* = 0.57), and the fixed effect model was used for analysis. The results showed that after intervention, the VO_2peak_ of patients in the experimental group was higher than that in the control group, the difference was statistically significant (MD = 1.85, 95% CI: 0.13 to 3.57, *p* = 0.04) (Fig. 2A). The quality of the evidence for this outcome according to the GRADE guidelines was moderate, considering imprecision as a factor that rating down.

### Study outcomes of 6-minute walking test

**Fig. 3:**
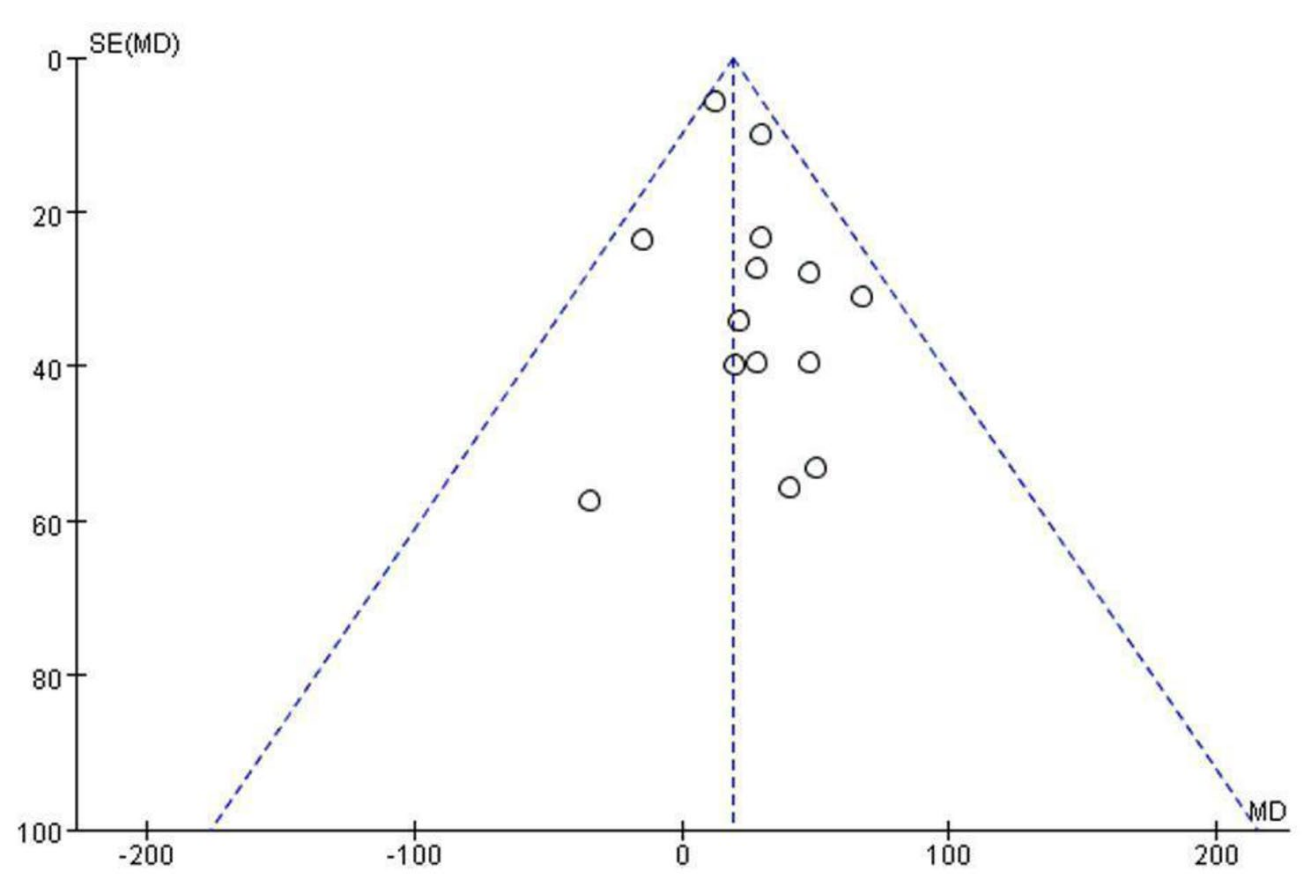
Funnel plot from studies included in the 6MWT

Overall, fourteen studies [13, 21–30, 32–34] evaluated baseline data and post-treatment results of the 6MWT in 582 patients. The heterogeneity between the studies was low (I^2^ = 0%, *p* = 0.70), and the fixed effect model was used. The results showed that the MD of gait training was 19.26 (95% CI: 10.43 to 28.08), which was superior to that of the control group (z = 4.28, *p* < 0.0001) (Fig. 2B). The funnel map analysis of 14 articles with the outcome index of 6MWT [13, 21–30, 32–34] showed that most of the studies were at the top and that the funnel map was not very symmetrical on both sides, so there may be some risk of bias (Fig. 3). The quality of the evidence for this outcome according to the GRADE guidelines was moderate, considering risk of bias as a factor that rating down.

### Study outcomes of peak heart rate

**Fig. 4:**
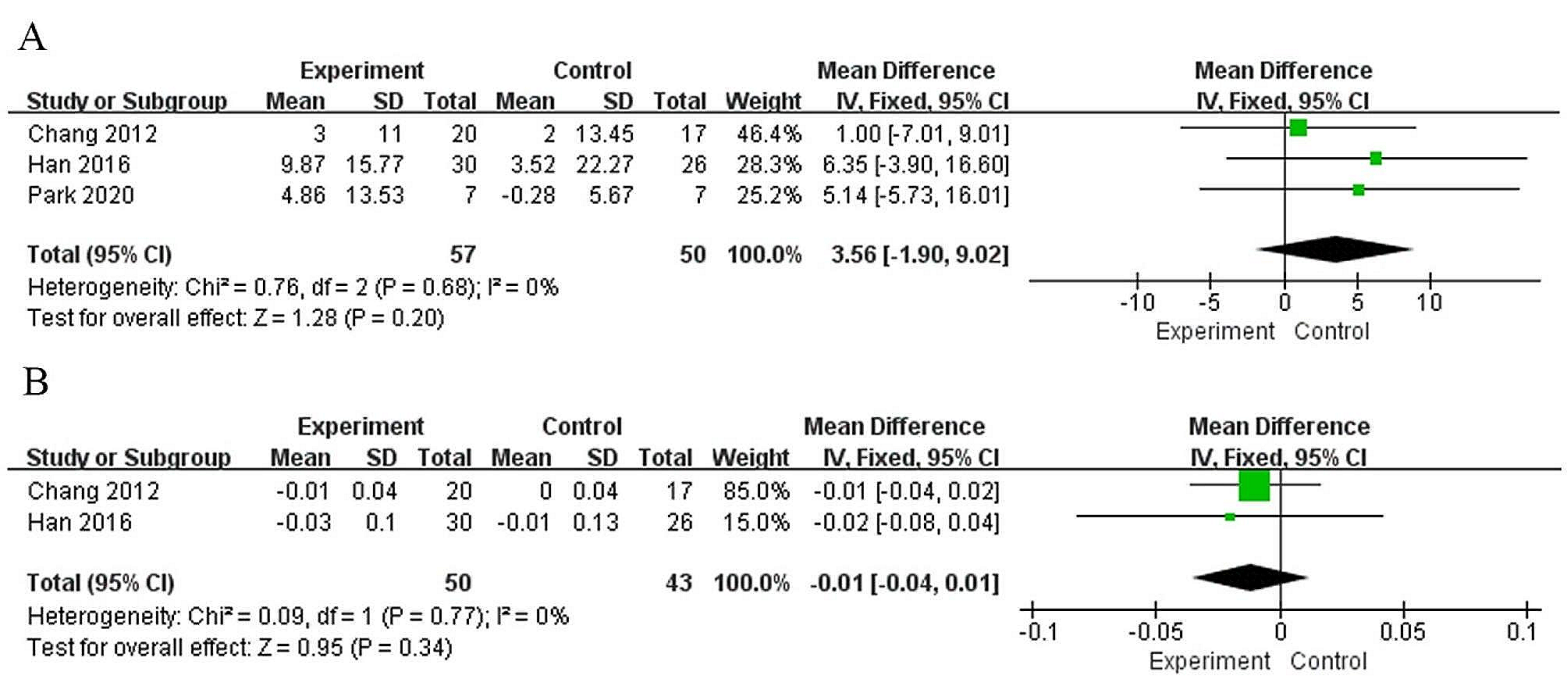
(**A**) Forest plots of meta-analysis to evaluate the effects of robot-assisted gait training on HR_peak_; (**B**) Forest plots of meta-analysis to evaluate the effects of robot-assisted gait training on RER_peak_

A total of 3 included studies [14, 31, 35] including 107 patients used HR_peak_ as an outcome measure. There was no obvious heterogeneity between the studies (I^2^ = 0%, *p* = 0.68), and the fixed effect model was used for analysis. The results showed that the HR_peak_ of patients in the experimental group was higher than that in the control group after intervention, but the difference was not statistically significant (MD = 3.56, 95% CI: -1.90 to 9.02, *p* = 0.20) (Fig. 4A). The quality of the evidence for this outcome according to the GRADE guidelines was low, considering risk of bias and imprecision as factors that rating down.

### Study outcomes of peak inspiratory expiratory ratio

Only 2 studies [31, 35] compared the improvement in RER_peak_ in 93 patients. Heterogeneity among studies was high (I^2^ = 0%, *p* = 0.77), and a fixed effect model was used. The results showed that there was no statistically significant difference between the experimental group and the control group in the improvement of RER_peak_ in stroke patients (MD = -0.01, 95% CI: -0.04 to 0.01, *p* = 0.34) (Fig. 4B). The quality of the evidence for this outcome according to the GRADE guidelines was moderate, considering imprecision as a factor that rating down.

### Risk of bias

**Fig. 5:**
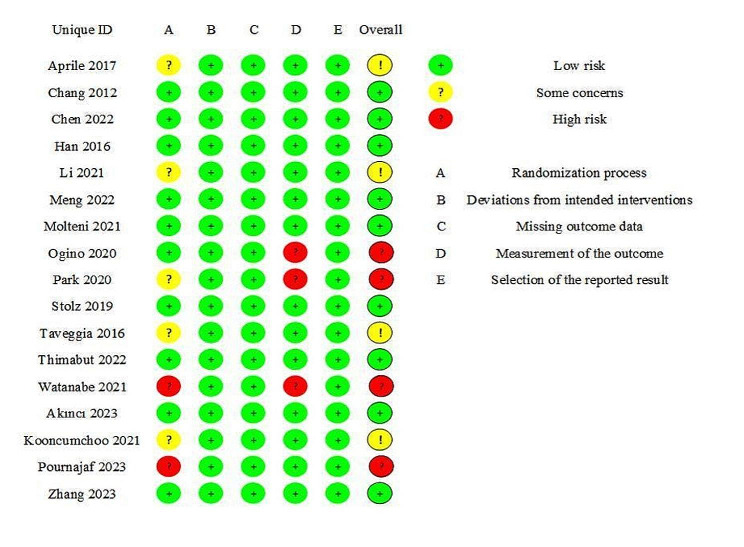
Risk of bias for the randomized controlled trials

The literature quality of the 17 included studies were evaluated in the systematic review and meta-analysis. The two investigators who assessed the risk of bias (CX and MSJ) agreed on 88% of the domains. The results of 9 studies were rated as low risk of bias [13, 21, 23, 25, 26, 30, 31, 34, 35], 4 studies were rated as some concerns [27, 28, 32, 33], and the remaining four were rated as high risk of bias [14, 22, 24, 29] (Fig. 5).

## Discussion

A total of 17 recent moderate and high-quality randomized controlled trials were included, extracting the intervention parameters (treatment type, training time, weekly frequency, training period), conducting data synthesis and analysis of primary outcome, mainly including VO2peak, 6 MWT, HRpeak and RERpeak. The results showed that robot-assisted gait training had significant positive effects on both 6MWT and VO_2peak_. There was no significant difference between treatment and control groups in HR_peak_ and RER_peak_. This study showed that robot-assisted gait training is beneficial for early cardiovascular function after stroke with high safety, providing reference for future related research and clinical practice.

As an objective indicator to assess cardiopulmonary health, VO_2peak_ reflects the maximum oxygen uptake achieved by the participants through maximum efforts. The measurement of VO_2peak_ using CPET is generally considered as the gold standard of cardiopulmonary capacity [36]. Only 2 articles [31, 35] in the current meta-analysis evaluated the short-term effects of robot-assisted gait training on VO2peak in stroke patients. The combined data showed that MD increased by 1.85 between treatment and control groups, which was statistically significant and achieved a minimum clinically important difference (MCID) value of 1 mL/kg/min [37]. Although the improved oxygen uptake after cardiovascular training appeared to be small, a slight increase in aerobic capacity may mean the difference between dependence and independence in all activities of daily living, given that stroke patients require higher aerobic capacity to maintain basic ADL function due to limb dysfunction [38, 39]. Of the included studies, fourteen [13, 21–30, 32–34] were available for quantitative assessment of the short-term effect of robot-assisted gait training on 6MWT in stroke patients. There was statistically significant effect between the treatment and control groups, with an overall MD difference of 19.26. It shows that robot-assisted gait training can improve the 6 min walking distance of stroke patients, and the improvement of walking ability suggests the enhancement of aerobic exercise ability, which may help to increase the training participation of stroke patients and enhance activities of daily living. But the changes induced during a 6-minute walk were not enough to achieve the smallest clinically significant difference of 34.4 m, so more studies are needed to confirm this positive effect [40]. However, according to the GRADE guideline, the evidence quality of VO2peak and 6MWT is medium, and the results of the combined analysis may be altered.

HRpeak is useful for estimating cardiovascular relative stress response during exercise [41]. Three studies [14, 31, 35] using HRpeak were selected for data synthesis analysis. Although the difference between the groups was not statistically significant, there was a significant clinical improvement in the robot-assisted gait training group compared to the minimum clinically significant difference of 1.46 beats/min [14]. However, for respiratory exchange ratio (RERpeak) assessment of aerobic capacity, the synthetic analysis of the two results [31, 35] showed that there was no significant overall difference between the treatment group and the control group, suggesting that robot-assisted gait training is of uncertain effectiveness in improving RERpeak in patients with stroke. Most of the studies included in this meta-analysis selected participants in the subacute and convalescent phase 2 weeks after the onset of stroke. All studies had strict exclusion criteria and did not consider stroke patients with severe heart disease. None of the studies reported major adverse events directly attributable to cardiovascular training. It is suggested that early cardiovascular rehabilitation training after stroke is relatively safe. It also raises the possibility of starting cardiopulmonary exercise training and testing in the early stage after stroke.

There are several limitations to our meta-analysis. First of all, the results of this study are not applicable to the general stroke population, given that except for one study [13] that included stroke patients within 48 h of the onset of the disease, all the other studies included patients in the subacute stage and recovery stage of stroke. Future studies need to develop reliable methods to assess aerobic capacity in individuals with severe disabilities and in the acute post-stroke period. Secondly, components of stroke rehabilitation program include frequency, intensity, time and type, which are known as FITT principles, and these components play a decisive role in the influence of exercise on CRF and walking ability [42].

Previous studies have shown that the most common exercise regimen is 30 to 40 min per exercise 3 times per week for 8 to 12 weeks [34], while this study included a relatively short intervention duration (2w − 6w). And due to the small number of studies included in the meta-analysis and limited data available for quantitative analysis, the fact that these studies can only be combined with short-term effects may be considered a limitation. Future studies need to continue to focus on the long-term effects of early stroke training interventions and the relationship between CRF improvement and daily living function in conjunction with more sensitive measures. Finally, the quality of these studies varies, assignment concealment, blinding of subjects and assessors were not explicitly stated or absent in the method, which interfered with the reliability of the research results.

## Conclusion

This meta-analysis demonstrates robot-assisted gait training may have a beneficial effect in improving VO2peak and 6WMT, with a moderate recommendation level according to the GRADE guidelines. Robot-assisted gait training as a new intervention measure for cardiopulmonary rehabilitation after stroke may be safe and feasible. Further research is needed to explore the effectiveness of early robotics in patients with different stages and degrees of stroke and to evaluate the long-term effects of robotics on aerobic capacity, physical function, and quality of life.

### Electronic supplementary material

Below is the link to the electronic supplementary material.

Supplementary Material 1

Supplementary Material 2

Supplementary Material 3

## Data Availability

The data collected in this study are available from the corresponding author on reasonable request. All primary data were extracted from the referenced sources.
