# Supplementary material for: Effect of robot-assisted gait training on improving cardiopulmonary function in stroke patients: a meta-analysis
Source: J Neuroeng Rehabil. 2024 May 30;21:92. doi: 10.1186/s12984-024-01388-9 (PMC11138000; doi:10.1186/s12984-024-01388-9)
Supplement: Supplementary file 1 — Supplementary Material 1 [file 12984_2024_1388_MOESM1_ESM.docx]

**Abbreviations**

RCT randomized controlled trail

CRF cardiorespiratory fitness

ADL activity of daily living

PRISMA reporting Items for Systematic Reviews and Meta-Analysis

CVA cerebralvascular accident

MD mean difference

CI confidence intervals

RAGT robot-assisted gait training

RRT routine rehabilitation therapy

baPWV brachial-ankle pulse wave velocity

VO2peak peak oxygen uptake

HRpeak peak heart rate

ETT exercise tolerance test

HRresting resting heart rate

6MWT 6-minute walk test

RERpeak peak inspiratory expiratory ratio

TWT the 10 m walking test

TUG timed up and go

SF-8 8-item short-form health survey

GRC global rating of change

FAC functional ambulation category

BBS berg balance scale

BRPE borg rating of perceived exertion

BDI-II beck depression inventory-II

ABC scale the activities-specific balance confidence scale

SPSR the swing phase symmetry ratio

SLSR step length symmetry ratio

MWS maximal walking speed

6MWD 6-minute walking distance

SPPB short physical performance battery

FMA-LE fugl-meyer assessment lower-limb subscale

FIM-WAK functional independence measure walk

EXP experimental group

CON control group;
