# Supplementary material for: Effect of robot-assisted gait training on improving cardiopulmonary function in stroke patients: a meta-analysis
Source: J Neuroeng Rehabil. 2024 May 30;21:92. doi: 10.1186/s12984-024-01388-9 (PMC11138000; doi:10.1186/s12984-024-01388-9)
Supplement: Supplementary file 2 — Supplementary Material 2 [file 12984_2024_1388_MOESM2_ESM.docx]

**Database retrieval strategy**

**Pubmed**

#1 = ((((((((stroke[MeSH Terms]) OR (cerebrovascular accident[MeSH Terms])) OR (CVA[MeSH Terms])) OR (cerebrovascular apoplexy[MeSH Terms])) OR (apoplexy[MeSH Terms])) OR (cerebral hemorrhage[MeSH Terms])) OR (intracerebral hemorrhage[MeSH Terms])) OR (cerebral infarction[MeSH Terms])) OR (brain infarction[MeSH Terms])

#2 = (((gait training[MeSH Terms]) OR (gait exercise[MeSH Terms])) OR (walking training[MeSH Terms])) OR (walking exercise[MeSH Terms])

#3 = ((((((cardiovascular reserve[Title/Abstract]) OR (6MWT[Title/Abstract])) OR (six-minute walk test[Title/Abstract])) OR (6-minute walk test[Title/Abstract])) OR (cardiorespiratory capacity[Title/Abstract])) OR (cardiopulmonary function[Title/Abstract])) OR (cardiopulmonary exercise test*[Title/Abstract])

#4 = (((randomized controlled trial[Publication Type]) OR (controlled clinical trial[Publication Type])) OR (clinical trial[Publication Type])) OR (randomized controlled trial[Title/Abstract])

#5 = #1 AND #2 AND #3 AND #4

**EMBASE**

#1 = stroke:ti,ab,kw OR 'cerebrovascular accident':ti,ab,kw OR cva:ti,ab,kw OR 'cerebrovascular apoplexy':ti,ab,kw OR apoplexy:ti,ab,kw OR 'cerebral hemorrhage':ti,ab,kw OR 'intracerebral hemorrhage':ti,ab,kw OR 'cerebral infarction':ti,ab,kw OR 'brain infarction':ti,ab,kw

#2 = 'gait training':ti,ab,kw OR 'gait exercise':ti,ab,kw OR 'walking training':ti,ab,kw OR 'walking exercise':ti,ab,kw

#3 = 'cardiovascular reserve':ti,ab,kw OR '6mwt':ti,ab,kw OR 'six-minute walk test':ti,ab,kw OR '6-minute walk test':ti,ab,kw OR 'cardiorespiratory capacity':ti,ab,kw OR 'cardiopulmonary function':ti,ab,kw OR 'cardiopulmonary exercise test*':ti,ab,kw

#4 = 'randomized controlled trial':ti,ab,kw OR 'controlled clinical trial':ti,ab,kw OR 'clinical trial':ti,ab,kw

#5 = #1 AND #2 AND #3 AND #4

**Web of science**

#1 = ((((((((TS=(stroke)) OR TS=(cerebrovascular accident)) OR TS=(cva)) OR TS=(cerebrovascular apoplexy)) OR TS=(apoplexy)) OR TS=(cerebral hemorrhage)) OR TS=(intracerebral hemorrhage)) OR TS=(cerebral infarction)) OR TS=(brain infarction)

#2 = (((TS=(gait training)) OR TS=(gait exercise)) OR TS=(walking training)) OR TS=(walking exercise)

#3 = ((((((TS=(cardiovascular reserve)) OR TS=(6mwt)) OR TS=(six-minute walk test)) OR TS=(6-minute walk test)) OR TS=(cardiorespiratory capacity)) OR TS=(cardiopulmonary function)) OR TS=(cardiopulmonary exercise test*)

#4 = ((TS=(randomized controlled trial)) OR TS=(controlled clinical trial)) OR TS=(clinical trial)

#5 = #1 AND #2 AND #3 AND #4

**Cochrane Database of Systematic Reviews**

#1 = (stroke):ti,ab,kw OR (cerebrovascular accident):ti,ab,kw OR (cva):ti,ab,kw OR (cerebrovascular apoplexy):ti,ab,kw OR (apoplexy):ti,ab,kw OR (cerebral hemorrhage):ti,ab,kw OR (intracerebral hemorrhage):ti,ab,kw OR (cerebral infarction):ti,ab,kw OR (brain infarction):ti,ab,kw (Word variations have been searched)

#2 = (gait training):ti,ab,kw OR (gait exercise):ti,ab,kw OR (walking training):ti,ab,kw OR (walking exercise):ti,ab,kw (Word variations have been searched)

#3 = (cardiovascular reserve):ti,ab,kw OR (6mwt):ti,ab,kw OR (six minute walk test):ti,ab,kw OR (6 minute walk test):ti,ab,kw OR (cardiorespiratory capacity):ti,ab,kw OR (cardiopulmonary function):ti,ab,kw OR (cardiopulmonary exercise test*):ti,ab,kw (Word variations have been searched)

#4 = (randomized controlled trial):ti,ab,kw OR (controlled clinical trial):ti,ab,kw OR (clinical trial):ti,ab,kw (Word variations have been searched)

#5 = #1 AND #2 AND #3 AND #4

**CNKI**

((((( 或者 (题名/关键词/摘要=步态训练 或者 V_SUBJECT=步态训练)) 或者 (题名/关键词/摘要=步态锻炼 或者 V_SUBJECT=步态锻炼)) 或者 (题名/关键词/摘要=步行训练 或者 V_SUBJECT=步行训练)) 或者 (题名/关键词/摘要=步行锻炼 或者 V_SUBJECT=步行锻炼)) 并且 ((((( 或者 (题名/关键词/摘要=脑卒中 或者 V_SUBJECT=脑卒中)) 或者 (题名/关键词/摘要=中风 或者 V_SUBJECT=中风)) 或者 (题名/关键词/摘要=脑血管意外 或者 V_SUBJECT=脑血管意外)) 或者 (题名/关键词/摘要=脑出血 或者 V_SUBJECT=脑出血)) 或者 (题名/关键词/摘要=脑梗死 或者 V_SUBJECT=脑梗死)) 并且 (题名/关键词/摘要=心肺) 或者 (题名/关键词/摘要=呼吸 或者 V_SUBJECT=呼吸)))(精确匹配); 数据库：中国医院知识总库(CHKD) 跨库检索

**Wangfang**

((主题=心肺) OR 主题=呼吸) AND ((((主题=步态训练) OR 主题=步态锻炼) OR 主题=步行训练) OR 主题=步行锻炼) AND (((((主题=脑卒中) OR 主题=中风) OR 主题=脑血管意外) OR 主题=脑出血) OR 主题=脑梗死)

**CBM**

("心肺"[常用字段:智能] OR "呼吸"[常用字段:智能]) AND ("步态训练"[常用字段:智能] OR "步态锻炼"[常用字段:智能] OR "步行训练"[常用字段:智能] OR "步行锻炼"[常用字段:智能]) AND ("脑卒中"[常用字段:智能] OR "中风"[常用字段:智能] OR "脑血管意外"[常用字段:智能] OR "脑出血"[常用字段:智能] OR "脑梗死"[常用字段:智能])
