# Supplementary material for: Effect of robot-assisted gait training on improving cardiopulmonary function in stroke patients: a meta-analysis
Source: J Neuroeng Rehabil. 2024 May 30;21:92. doi: 10.1186/s12984-024-01388-9 (PMC11138000; doi:10.1186/s12984-024-01388-9)
Supplement: Supplementary file 3 — Supplementary Material 3 [file 12984_2024_1388_MOESM3_ESM.docx]

**data extraction form**

**Table 1.** Demographic characteristics of the randomized clinical trials (n=17) that were included in the meta-analysis

| **Study** | **Country of study** | **Sample size experimental /control group(n)** | **Age (years)** | **Sex (F/M)** | **Disease duration(days)** | **Course of disease** |
| --- | --- | --- | --- | --- | --- | --- |
| Han et al.^[35]^  (2016) | Republic of Korea | 30/26 | experimental: 67.89±14.96 control: 63.2±10.62 | 24/32 | experimental: 21.56±7.98 control: 18.10±9.78 | <3 months after stroke |
| Chang et al.^[31]^  (2012) | Republic of Korea | 20/17 | experimental: 55.5(12.0,27-76) control :59.7(12.1,37-79) | 14/23 | experimental :16.1(4.9) control :18.2(5.0) | ≤1 month after stroke |
| Li et al.^[32]^  (2021) | China | 17/15 | experimental: 50.53(12.26) control: 50.13(9.49) | 3/29 | experimental: 2.53(1.33) control: 3.38(1.19) (month) | from 2 weeks to 6months |
| Taveggia et al.^[33]^  (2016) | Italy | 13/15 | experimental: 71±5 control: 73±7 | 11/17 | experimental: 60.1±49.5 control: 39.4±31.7 | < 6 months after stroke |
| Ogino et al.^[22]^  (2020) | Japan | 8/11 | experimental: 66.1(9.6) control:65.0(7.7) | 4/15 | experimental: 8.0(5.1)  control: 7.1(4.1) (year) | >6 months after stroke |
| Park et al.^[14]^  (2020) | Republic of Korea | 7/7 | experimental: 76.29 control: 69.86 | 5/9 | 2-weeks post-stroke onset | 2-weeks post-stroke |
| Molteni et al.^[23]^  (2021) | Italy | 38/37 | experimental: 62.13±8.75 control: 68.24±8.58 | 36/42 | experimental: 35.68±10.70 control: 34.14±16.07 | ≤6 months after stroke |
| Watanabe et al.^[24]^  (2021) | Japan | 9/11 | experimental: 60.0±11.7 control: 77.4±14.3 | 10/10 | experimental: 60.7±50.7 control: 46.5±34.4 | subacute-to-recovery stroke |
| Chen et al.^[25]^  (2022) | China | 29/29 | experimental: 48.86±11.96 control: 50.86±11.06 | 14/44 | experimental: 3.48±1.30 control: 3.45±1.24 (month) | from 2 weeks to 6 months |
| Meng et al.^[13]^  (2022) | China | 62/61 | experimental: 59.36±1.65 control: 60.12±1.73 | 55/68 | Na | ≤48 h after stroke |
| Thimabut et al.^[26]^  (2022) | Thailand | 13/13 | experimental: 52.8±12.6 control: 62.8±8.5 | 10/16 | experimental: 56.15±23.71 control: 72.54±20.12 | ≤90 d after stroke |
| Aprile et al.^[27]^  (2017) | Italy | 6/8 | Experimental: 58±20.74 control: 69.12±10.51 | 5/9 | Na | >6 months after onset |
| Stolz et al.^[34]^  (2019) | Australia | 20/16 | experimental: 68.1(12.5) control: 63.2(16.6) | 14/22 | experimental: 15.6(9.4)  control: 25.5(21.8) | < 3 months after stroke onset |
| Akıncı et al.^[21]^  (2023) | Turkey | 14/14 | experimental:57.71±6.41  control:64.07±5.54 | 0/28 | experimental:237.21±28.91  control:259.07±56.13 | >6 months after stroke onset |
| Kooncumchoo et al.^[28]^  (2021) | Thailand | 15/15 | experimental:64.33±7.68  control:63.53±12.16 | 10/20 | experimental:6.57±3.57  control:5.60±5.65 (year) | >6 months after stroke onset |
| Pournajaf et al.^[29]^  (2023) | Italy | 31/28 | experimental:59.42±15.51  control:63.39±12.85 | 23/36 | experimental:63.68±49.92  control:58.46±43.24 | < 6 months after stroke onset |
| Zhang et al.^[30]^  (2023) | China | 18/16 | experimental:56.88±10.99  control:60.81±9.61 | 7/27 | experimental:2.5±4.00  control:3.5±3.00 (month) | < 6 months after stroke onset |

**Table 2.** Information on interventions of the included studies

| **Study** | **Intervention/control type** | **Training time** | **Weekly frequency** | **Training period (weeks)** | **Primary outcome** | **Adverse event** | **Outcomes measures** |
| --- | --- | --- | --- | --- | --- | --- | --- |
| Han et al.^[35]^  (2016) | RAGT+RRT/RRT | 30 min | 5 times | 4 | baPWV, VO_2peak_,  HR_peak_, ETT duration | Na | pre-test, post-test |
| Chang et al.^[31]^  (2012) | RAGT+RRT/RRT | 40 min | 5 times | 2 | VO2peak, RER_peak_,  HR_resting_, Hr_peak_ | Na | pre-test, post-test |
| Li et al.^[32]^  (2021) | RAGT/RRT | 30 min twice a day | 5 times | 4 | 6MWT | No | pre-test, post-test, 3 months |
| Taveggia et al.^[33]^  (2016) | RAGT+RRT/RRT | 30 min | 5 times | 5 | 6MWT, TWT | No | week 0, week 5, week 17 |
| Ogino et al.^[22]^  (2020) | RAGT/RRT | 40 min | 5 times | 4 | TWT, TUG, 6MWT,  SF-8, GRC | No | pre-test, post-test, week 8, 3 months |
| Park et al.^[14]^  (2020) | RAGT/RRT | 30 min | 7 times | 2 | FAC, BBS, HR, BRPE, BDI-II, ABC, SCALE | Na | pre-test, post-test |
| Molteni et al.^[23]^  (2021) | RAGT/RRT | 60 min | 5 times | 3 | 6MWT | No | pre-test, post-test |
| Watanabe et al.^[24]^  (2021) | RAGT/RRT | 20 min | 3 times | 4 | FAC, MWS, step length, cadence, 6MWD, SPPB, FMA-LE | Na | pre-test, post-test |
| Chen et al.^[25]^  (2022) | RAGT+RRT/RRT | 30 min twice a day | 5 times | 2/4 | 6MWT, FAC,  FMA-LE | Na | week 0, week 2, week 4 |
| Meng et al.^[13]^  (2022) | RAGT/RRT | 45 min | 3 times | 4 | 6MWT, FAC | No | pre-test, post-test |
| Thimabut et al.^[26]^  (2022) | RAGT+RRT/RRT | 30 min | 5 times | 6 | FIM-WAK score,  efficacy of FIM-WAK | Na | baseline, treatment 15th, treatment 30th |
| Aprile et al.^[27]^  (2017) | RAGT /RRT | 45 min | 3 times | 6.5 | Motricity Index, Ashworth Scale, MRC, Timed Up and Go Test, 6MWT, Ten-Meter Walk Test, FAC, W.H.S, Tinetti Scale, FMA, Trunk Control Test. BTS | Na | before treatment and at the end of the rehabilitation program |
| Stolz et al.^[34]^  (2019) | RAGT /RRT | 60 min | 5 times | average 3 | 10MWT | Na | on admission, discharge, and 4 weeks post discharge |
| Akıncı et al.^[21]^  (2023) | RAGT+RRT/RRT | 40 min | 3 times | 6 | H-SL, non-H-SL, SL, SW, H-SP, non-H-SP, DSP, 6MWT, 10MWT, BBS | Na | pre-test, post-test |
| Kooncumchoo et al.^[28]^ (2021) | RAGT+RRT/RRT | 60min | 3 times | 8 | 6MWT, 10MWT, Timed Up and Go Test | Na | week0, week2, week4, week6, week8 |
| Pournajaf et al.^[29]^  (2023) | RAGT/RRT | 30min | 3-5times | 4-6.5 | 10MWT | No | pre-test, post-test |
| Zhang et al.^[30]^  (2023) | RAGT/RRT | 30min | 5 times | 4 | Spatiotempora, kinematic parameters | Na | pre-test, post-test |
